# Liver Transplantation in the Presence of Portal Vein Thrombosis

**DOI:** 10.1155/1992/83425

**Published:** 1992

**Authors:** Paul McMaster

**Affiliations:** Liver and Hepatobiliary Unit Queen Elizabeth Hospital Edgbaston Birmingham B15 2TH UK


*HPB Surgery*, 1992, Vol. 5, pp. 217–219
Reprints available directly from the publisher
Photocopying permitted by license only

© 1992 Harwood Academic Publishers GmbH
Printed in the United Kingdom

### HPB INTERNATIONAL

EDITORIAL & ABSTRACTING SERVICE

JOHN TERBLANCHE, EDITOR
Department of Surgery,
Medical School · Observatory 7925
Cape Town · South Africa
Telephone: (021) 47-1250 Ext. 2
Telefax (021) 448-6461

### LIVER TRANSPLANTATION IN THE PRESENCE OF PORTAL VEIN THROMBOSIS

### ABSTRACT

*Stieber, A.C., Zetti, G., Todo, S., Tzakis, A.G., Fung, J.J., Marino, I., Casavilla, A., Selby, R.R. and Starzl, T.E. (1991) The spectrum of portal vein thrombosis in liver transplantation. Annals of Surgery*; **213**: 199–206

**Thrombosis of the portal vein with or without patency of its tributaries used to be a contraindication to orthotopic liver transplantation (OLTX) until quite recently. Rapid progress in the surgical technique of OLTX in the last few years has demonstrated that most patients with portal vein thrombosis can be safely and successfully transplanted. Presented here is a series of thirty-four patients with portal vein thrombosis transplanted at the University of Pittsburgh since 1984. The various techniques used to treat various forms of thrombosis are described. The survival rate for this series was 67.6% (23 of 34 patients). Survival was best for patients who underwent phlebothrombectomy or placement of a jump graft from the superior mesenteric vein. The survival rate also correlated with the amount of blood required for transfusion during surgery. Overall it is concluded that a vast majority of the patients with thrombosis of the portal system can be technically transplanted and that their survival rate is comparable to that of patients with patient portal vein.**

### PAPER DISCUSSION

KEY WORDS: Liver transplantation, portal vein thrombosis

The authors of the worlds most active liver transplant centre have summarised their experience of transplantation in recipients with portal vein thrombosis, something which was long considered to be a specific contra-indication. They review the treatment options for dealing with such a situation and outline broadly the very successful conclusions.

Difficulties with the portal vein in the recipient can occur broadly in two situations. There is firstly, the hypoplastic portal vein in children with longstanding biliary atreasia which fails to provide adequate perfusion of the implanted graft[1]. Although not a true thrombosis, the technical reconstructions of conduits and grafts are almost invariably needed if adequate flow is to be achieved. But, secondly, and more commonly, there is the situation in the recipient in whom a thrombosis of the portal vein has occurred and sometimes additionally of the superior mesenteric veins. Faced with that situation, there are probably two prime options. Firstly, the removal of the thrombus from within the vessels, thrombectomy, has been undertaken and as the authors indicate is usually sucessful, particularly when a very short segment of portal vein is involved. Indeed, the authors commendation of thrombectomy as the most appropriate way of dealing with the situation alludes to the fact that the majority of thrombosis are of relatively short extent. However, more extended thrombi, particularly when longstanding, cannot be as easily cleared and then one of the vascular reconstructions are needed. The standard alternative of venous grafting through the transverse meso-colon to the superior mesenteric vein, may need to be augmented by more innovative techniques. Reconstruction to an enlarged coronary vein or indeed, massive gastric varices have been described,[2,3], and the crucial component is the establishment of adequate portal flow. A flow of between 1 and 1.5 litres a minute is probably adequate for the newly implanted liver[4], which can be particularly vulnerable to hypoxia and hypoperfusion. It is perhaps worth recording that while hepatic artery ligation or thrombosis in normal circumstances is relatively well tolerated, in a transplant patient with a newly implanted graft, thrombosis, of either hepatic artery or portal vein may lead to rapid failure of the newly implanted graft that is bereft of collaterals[5].

Perhaps an area which does require highlighting is the aditional time this complex reconstructive procedure adds to the operative time to implant the organ. Once the graft is in place, the graft warming process begins and normal anastomotic times of 30–40 minutes are recorded. However, if complex vascular reconstructions are needed, this period of time can be extended significantly and may lead to primary dysfunction with an increased risk of graft failure. One way of addressing this issue is to undertake the reconstruction in the recipient with the conduit placed to the superior mesenteric vein and splenic vein before the graft liver is implanted, so that the graft liver can go straight on to the implanted venous conduit[1].

Perhaps what is much less clear, is the value of anti-thrombotic agents after such complex reconstruction procedured. While Aspirin, Dipyridamole, Heparin and Dextran have all been advocated, the objective evidence that they are of value is far from clear.

One particular circumstance, however, which does clearly require careful coagulation monitoring is the Budd-Chiari syndrome which, not uncommonly, has secondary thrombosis in the splenic or portal vein. Under those circumstances not only does the primary condition, often Polycythemia Rubra Vera, require to be addressed, but subsequent anti-coagulation initially with Heparin and long term Warfarin will be needed.

In summary, therefore, the authors wide experience of grafting in the presence of portal vein thrombosis (some 9.7% of transplant recipients) highlights clearly the technical options in dealing with such an eventuality. That patients with multiple thrombosis can be grafted, is not in doubt, but the increased technical difficulties and time involved are a significant additional risk which requires careful evaluation.

### REFERENCES

1. Shaw, B.W. Jnr., Iwatsuki, S., Bron, K. *et al.* (1985) Portal vein grafts in hepatic transplantation. *Surg. Gynaecol. Obstet.* **161**, 66–68
2. Cherniak, A., Badger, I. and Buckels, J. (1990) Orthotopic liver transplantation in a patient with thrombosis of the hepatic portal and superior mesenteric vein. *Transplantation,* **50**, 334–335
3. Hiat, J.R., Quinonees-Baldrich, W.J., Ramming, K.P. *et al.* (1986) Bile duct varices: an alternative to porto-portal anastomosis in liver transplantation. *Transplantation,* **42**, 85
4. Kirsch, J.P., Howard, T.K., Klintmalm, G.B. *et al.* (1990) Problematic vascular reconstruction in liver transplantation, Part 11. Porto-venous conduits. *Surgery,* **107**, 544–548
5. Starzl, T.E. and Demetris, A.J. (1990) *Liver transplantation,* pp. 45–49 Year Book Medical Publishers, Chicago, London Boc & Paton, Littleton, Mass.

Paul McMaster
Consultant Surgeon
Liver and Hepatobiliary Unit
Queen Elizabeth Hospital
Edgbaston
Birmingham, B15 2TH
United Kingdom

